# Eineinhalb Jahre E-Scooter – Zwischenbilanz in Hamburg: Teil 2: Unfallcharakteristika

**DOI:** 10.1007/s00194-022-00602-z

**Published:** 2023-01-05

**Authors:** Antonia Kähler, Klaus Püschel, Benjamin Ondruschka, Darius Thiesen, Holger Kleinertz, Antonia Fitzek

**Affiliations:** 1 Institut für Rechtsmedizin Hamburg, Universitätsklinikum Hamburg-Eppendorf, Butenfeld 34, 22529 Hamburg, Deutschland; 2 Klinik und Poliklinik für Unfallchirurgie und Orthopädie, Universitätsklinikum Hamburg-Eppendorf, Hamburg-Eppendorf, Deutschland

**Keywords:** E‑Scooter, Elektro-Scooter, Frakturen, Verkehrsunfall, Schädel-Hirn-Trauma, E‑scooters, Electric scooters, Fractures, Traffic accident, Craniocerebral trauma

## Abstract

**Hintergrund:**

Seit der Verbreitung der sog. Elektro-Scooter durch Verleihservices in Hamburg ab Juni 2019 sind Unfälle durch die Nutzung dieser Fortbewegungsmittel regelmäßig registriert worden. Die häufigsten Verletzungsmuster bei verunfallten E‑Scooter-Fahrern betrafen die obere Extremität und den Kopf. Es zeigte sich dabei eine relevante Anhäufung alkoholisierter Fahrer unter den verletzten Personen.

**Ziel der Arbeit:**

Ziel der vorliegenden Studie ist der Vergleich zwischen den Unfallmustern alkoholisierter und nichtalkoholisierter E‑Scooter-Fahrer.

**Material und Methoden:**

Die Daten der Unfallchirurgie des Universitätsklinikums Hamburg-Eppendorf wurden retrospektiv zu Unfällen mit E‑Scootern und hieraus resultierenden Verletzungsmustern ausgewertet. Hierfür wurden das Geschlecht, das Alter, das Verletzungsmuster der Personen, der Unfallhergang sowie Informationen über einen vorangegangenen Alkoholkonsum deskriptiv für den Zeitraum Juni 2019 bis Dezember 2021 erfasst.

**Ergebnisse:**

Die Fahrer der Gesamtkohorte waren durchschnittlich 32 (Intervall: 15 bis 88 Jahre) Jahre alt und diejenigen unter Alkoholeinfluss überwiegend männlich (69,9 %). Unfälle fanden v. a. im Sommer und nachts statt. Häufige Verletzungsmuster waren Verletzungen des Gesichts, des Kopfes und der Extremitäten.

**Schlussfolgerung:**

Festzuhalten ist, dass unter Alkoholeinfluss häufiger Verletzungen des Gesichts und des Kopfes vorlagen als bei Nüchternheit. Die Sensibilität für gesundheitliche und rechtliche Folgen von E‑Scooter-Fahrten unter Alkoholeinfluss muss verbessert werden. Zudem stellen eine Helmpflicht oder nächtliche Fahrverbote mögliche Maßnahmen zur Reduktion von Unfällen mit E‑Scootern dar.

## Abstract (en)

**Background:**

Since the spread of the so-called electric scooters (e-scooters) through rental services in Hamburg as of June 2019, accidents due to the use of this means of transportation have been regularly registered. The most frequent injury patterns among e‑scooter riders involved accidents concerning the upper extremities and the head. There was a relevant accumulation of drunk drivers among the injured persons.

**Objective:**

The aim of the study is to compare injury patterns in drunk versus non-drunk e‑scooter riders who had an accident.

**Material and methods:**

Data from the Department of Trauma Surgery at the University Medical Center Hamburg-Eppendorf were retrospectively analyzed for accidents involving e‑scooters and the resulting injury patterns. For this purpose, the gender, age, injury pattern of the persons, the course of the accident, and information on previous alcohol consumption were recorded descriptively for the period from June 2019 to December 2021.

**Results:**

Drivers were on average 32 (interval: 15–88 years) years old and those under the influence of alcohol were predominantly male (69.9%). Accidents occurred mainly in summer and at night. Common injury patterns were injuries to the face, head, and extremities.

**Conclusion:**

It should be noted that injuries to the face and head occurred more frequently under the influence of alcohol than in the cohort without alcohol. Education about the health and legal consequences of riding an e‑scooter under the influence of alcohol should be provided to all users. In addition, wearing a helmet or banning driving at night are possible measures to reduce accidents associated with drunk riding.

## Einführung

Seitdem am 21.06.2019 der Verleih von E‑Scootern durch Sharing-Anbieter in Hamburg startete, wurden verunfallte E‑Scooter-Fahrer in den zentralen Notaufnahmen wiederholt vorstellig.

E‑Scooter sind akkubetriebene Tretroller mit Elektromotor, die bis zu 20 km/h schnell werden können; sie dürfen ohne Führerschein gefahren und bereits ab 14 Jahren bedient werden [2, 25]. Die E‑Scooter-Sharing-Dienste in Großstädten wurden unter der Prämisse eingeführt, das „Problem der letzten Meile“ im urbanen Kontext zu lösen. Dies bedeutet, dass sie u. a. Pendlern ermöglichen, zeitsparend das letzte Stück des Weges zwischen Stationen des öffentlichen Nahverkehrs und dem Ziel zurückzulegen [3]. Die Dienste ermöglichen ein Entleihen der geparkten E‑Scooter per Smartphone-App, welche ohne komplizierten Registrierungsaufwand und ohne Fahrtraining für jedermann möglich ist [10]. Mittlerweile sind die E‑Scooter in den meisten Großstädten ubiquitär vorhanden und können ohne Parkaufwand fast überall abgestellt werden [4]. Der Fahrer steht mit einem Fuß vor dem anderen auf dem Brett; vor ihm befindet sich ein Lenker; mit einem Fuß wird Schwung geholt, und dann werden Gas und Bremse mit Hebeln am Lenker betätigt [24]. Die wissenschaftliche Datenlage zeigt, dass Unfälle mit E‑Scooter-Fahrern neuartige und gleichzeitig zunehmend relevante Vorkommnisse sind [1–3, 5, 8–12, 16, 17, 20, 21]. Dies begründet die Notwendigkeit einer ausführlichen Analyse der Einflussfaktoren, um das Risiko von Unfällen und einer damit einhergehenden Verletzung zu vermindern. Bis dato zeigen veröffentlichte Artikel typische Verletzungsmuster und Unfallmechanismen dieser E‑Scooter-Vorkommnisse auf [1–3, 5, 8–12, 16, 17, 20, 21]. Einige Autoren berichten dabei über einen relevanten Anteil intoxikierter Verunfallter [22, 24].

Ziel dieser Studie war es aus diesem Grund, einen Überblick über demografische Charakteristika sowie Verletzungsmuster verunfallter alkoholisierter E‑Scooter-Fahrer im Zeitraum Juni 2019 bis Dezember 2020 darzustellen und diese Daten mit denjenigen von nichtalkoholisierten Geschädigten zu vergleichen.

## Methodik

Für diese Studie wurden die elektronischen Krankenakten aus der Zentralen Notaufnahme und der Unfallchirurgischen Ambulanz des Universitätsklinikum Hamburg-Eppendorf retrospektiv mithilfe einer Schlagwortsuche analysiert. Erfasst wurden zunächst alle Patienten, welche im Zeitraum vom 21.06.2019 bis zum 31.12.2021 unter den Stichworten „Scooter“, „E-Scooter“, „Roller“ und „E-Roller“ zu finden waren. Eingeschlossen wurden alle Datenbanktreffer von Patienten, die zum Zeitpunkt des Unfallgeschehens aktiv mit einem E‑Scooter gefahren waren. Einzelne Fälle von verletzten Personen, die von einem E‑Scooter passiv angefahren wurden, sind in der Auswertung nicht berücksichtigt wurden.

Die Patientenakten dienten als Quelle für Informationen zum Alter, dem Geschlecht, anamnestischem oder gemessenem Alkoholkonsum zum Zeitpunkt des Unfalls, dem mitgeteilten Unfallzeitpunkt, dem Unfallhergang und den dokumentierten Verletzungen. Sofern keine klare Angabe zu ermitteln gewesen war, wurde dies als „keine Angabe“ verzeichnet.

Die statistische Analyse erfolgte deskriptiv mit Microsoft Excel (Version 16.16, Microsoft Corporation, Redmond, USA). Die Variablen wurden als Prozentsätze und Absolutzahlen beschrieben. Die Datensätze wurden vor Testung auf Normalverteilung überprüft. Kategoriale Variablen wurden mit dem Mann-Whitney-U-Test verglichen. Die grafische Darstellung der Ergebnisse erfolgte mit der Statistiksoftware GraphPad Prism® (Version 8.0, GraphPad Software Inc., La Jolla, USA). Zusätzlich wurde der Pearson-Chi-Quadrat-Test mit der Statistiksoftware GraphPad Prism® (Version 8.0, GraphPad Software Inc., La Jolla, USA) angewendet, um zu überprüfen, dass zwischen den kategorialen Variablen ein statistisch relevanter Zusammenhang besteht. Die grafische Darstellung der Ergebnisse erfolgte mit der gleichen Statistiksoftware GraphPad Prism®.

## Ergebnisse

Die Auswertung ergab 252 Fälle von verunfallten E‑Scooter-Fahrern. Von diesen trug anamnestisch eine Person zum Zeitpunkt des Unfalls einen Sturzhelm. Im Kollektiv wurde für 32,9 % (*n* = 83) der Patienten dokumentiert, dass sie vor dem Unfall anamnestisch Alkohol konsumiert hatten, während dieser bei 13,9 % (*n* = 35) explizit verneint wurde. Bei 53,2 % (*n* = 134) wurden keine Angaben hierzu gemacht. Nachfolgend wird die Kohorte der Verunfallten mit Alkoholkonsum „Kohorte 1“ (K1, *n* = 83) und die der Patienten ohne Alkoholkonsum „Kohorte 2“ (K2, *n* = 35) genannt.

### Alkoholisierte Verunfallte sind häufig Männer

**Figure 1:**
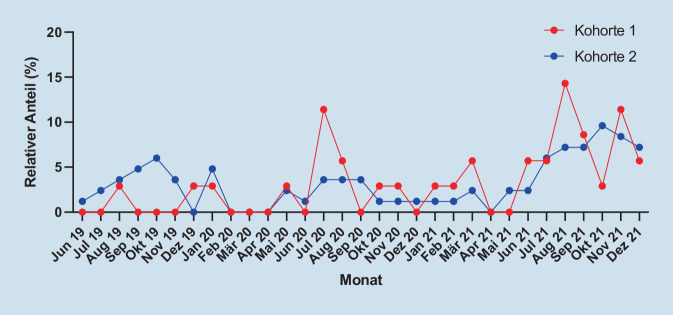

In K1 (*n* = 83) lag das Durchschnittsalter bei 31 Jahren (Median 30,3 Jahre), der jüngste Patient war 16, der älteste 60 Jahre alt, und 62,7 % (*n* = 52) waren über 25 Jahre alt. In dieser Kohorte waren 69,9 % (*n* = 58) und 30,1 % (*n* = 25) weiblich (Abb. 1). In K2 betrug das durchschnittliche Alter 32 Jahre (Median 32,2 Jahre), der jüngste Patient war 15 und der älteste 56 Jahre alt. 5,7 % (*n* = 2) der Patienten waren unter 18, 68,6 % (*n* = 24) über 25.

62,9 % (*n* = 22) der Verunfallten waren weiblich, 37,9 % (*n* = 13) männlich (Tab. 1).

**Table 1:** 

*Beschreibung*	*Kategorisierung*	*Unter C2-Einfluss (n* *=* *83)*	*Ohne C2-Einfluss (n* *=* *35)*
*Geschlecht*	Männlich Weiblich	58 (69,9 %) 25 (30,1 %)	13 (37,1 %) 22 (62,9 %)
*Alter (Jahre)*	< 18 18–25 26–40 41–64	2 (2,4 %) 29 (34,9 %) 38 (45,8 %) 14 (16,9 %)	2 (5,7 %) 9 (25,7 %) 18 (51,4 %) 6 (17,1 %)
*Unfallmechanismus*	Sturz Kollision bewegtes Objekt Kollision stehendes Objekt	72 (86,7 %) 6 (7,2 %) 5 (6,0 %)	29 (82,9 %) 4 (11,4 %) 2 (5,7 %)

Die Tabelle zeigt das Geschlecht, das Alter und den Unfallmechanismus in absoluten und relativen Werten. *C2* Alkohol

### Die meisten Verletzungen entstanden sturzbedingt

Die Unfallmechanismen in K1 wurden bei 86,7 % (*n* = 72) als Sturz, bei 6,0 % (*n* = 5) als Kollision mit stehenden Objekten und bei 7,2 % (*n* = 6) als Kollision mit anderen Verkehrsteilnehmern beschrieben.

Der Unfallmechanismus in K2 war in 82,9 % (*n* = 29) ein Sturz, in 5,7 % (*n* = 2) wurde die Verletzung durch eine Kollision mit einem stehenden Objekt verursacht und in 11,4 % (*n* = 4) durch eine Kollision mit anderen Verkehrsteilnehmern, zu je 2,4 % (*n* = 2) mit einem anderen E‑Scooter oder einem Pkw (Tab. 1).

### Alkoholisierte Fahrer haben eher Kopfverletzungen

Quetsch-Riss-Wunden des Gesichts waren bei 49,4 % (*n* = 41) in Kohorte 1 die häufigsten Verletzungen. Ebenfalls fazial kam es bei 24,1 % (*n* = 20) zu einer oder mehreren Frakturen des Gesichtsschädels. Eine Verletzung der Zähne wurde bei 34,9 % (*n* = 29) der Patienten festgestellt. Ein Schädel-Hirn-Trauma ersten Grades lag bei 22,9 % (*n* = 19) vor, eine intrakranielle Blutung bei 7,2 % (*n* = 6), davon jeweils in einem Fall 3 Kontusionsblutungen, ein Subduralhämatom und 2 traumatische Subarachnoidalblutungen. Die oberen Extremitäten waren bei 13,2 % (*n* = 11) von Hämatomen betroffen, die unteren bei 9,6 % (*n* = 8), in je einem Fall war die Clavicula, der Radius, der Humerus oder das Olecranon gebrochen. Jeweils eine Tibiakopffraktur, eine Fraktur des oberen Sprunggelenks, des Unterschenkels und der Patella traten ebenfalls singulär auf; Verletzungen des Abdomens wurden nicht festgestellt (Tab. 2).

**Table 2:** 

*Verletzungsort*	*Verletzungsart*	*Lokalisation*	*Anamnestisch unter C2-Einfluss (n* *=* *33)*		*Anamnestisch kein C2-Einfluss (n* *=* *83)*	
*Kraniofazial*	*Kopf/Gesicht*					
	Kontusion	–	17	20,5 %	5	14,3 %
	Schädel-Hirn-Trauma °1	–	19	22,9 %	1	2,9 %
	Schädel-Hirn-Trauma °3	–	1	1,2 %	0	0,0 %
	Schürfung	–	14	16,9 %	4	11,4 %
	Riss-Quetsch-Wunde	–	41	49,4 %	5	14,3 %
	*Fraktur*					
	–	Unterkiefer	4	4,8 %	0	0,0 %
	–	Nase	9	10,8 %	0	0,0 %
	–	Orbita	9	10,8 %	2	5,7 %
	–	Jochbein	3	3,6 %	1	2,9 %
	–	Oberkiefer	2	2,4 %	0	0,0 %
	–	Knöcherner Schädel	2	2,4 %	0	0,0 %
	Intrakranielle Blutung	–	3	3,6 %	0	0,0 %
*Obere Extremität*	*Kontusion*					
	–	Schulter	1	1,2 %	3	8,6 %
	–	Ellenbogen	1	1,2 %	4	11,4 %
	–	Hand	9	10,8 %	3	8,6 %
	Schürfung	–	4	4,8 %	7	20,0 %
	Luxation	Schulter	0	0,0 %	1	2,9 %
	Sprengung	Akromioklavikulargelenk	1	1,2 %	–	0,0 %
	*Fraktur*					
	–	Clavicula	1	1,2 %	0	0,0 %
	–	Humerus	1	1,2 %	3	8,6 %
	–	Radiuskopf	0	0,0 %	5	14,3 %
	–	Radius außer Radiuskopf	1	1,2 %	0	0,0 %
	–	Hand	5	6,0 %	2	5,7 %
	–	Olecranon	1	1,2 %	–	0,0 %
*Untere Extremität* *Untere Extremität*	*Kontusion*					
	–	Becken	2	2,4 %	1	2,9 %
	–	Hüfte	0	0,0 %	1	2,9 %
	–	Knie	6	7,2 %	3	8,6 %
	Riss-Quetsch-Wunde	–	2	2,4 %	0	0,0 %
	Schürfung	–	4	4,8 %	8	22,9 %
	*Distorsion*					
	–	Knie	1	1,2 %	1	2,9 %
	–	Oberes Sprunggelenk	1	1,2 %	4	11,4 %
	*Fraktur*					
	–	Tibiakopf	1	1,2 %	0	0,0 %
	–	Oberes Sprunggelenk	1	1,2 %	3	8,6 %
	–	Unterschenkel	1	1,2 %	0	0,0 %
	–	Patella	1	1,2 %	0	0,0 %
	*Kontusion*					
	–	Becken	2	2,4 %	1	2,9 %
*Zähne*	*Zahn*					
	Fraktur	–	19	22,9 %	1	2,9 %
	Luxation	–	6	7,2 %	1	2,9 %
	Avulsion	–	4	4,8 %	0	0,0 %
	Dentoalveoläres Trauma	–	3	3,6 %	0	0,0 %
*Thorax*	Kontusion	–	2	2,4 %	1	2,9 %
	Rippenfraktur	–	1	1,2 %	0	0,0 %
*Abdomen*	Milzlazeration	–	0	0,0 %	1	2,9 %
	Nierenkontusion	–	0	0,0 %	2	5,7 %
	Leberlazeration	–	0	0,0 %	1	2,9 %
*Wirbelsäule*	Distorsion	Halswirbelsäule	2	2,4 %	2	5,7 %

Die Tabelle zeigt die Arten der vorhandenen Verletzungen in absoluten und relativen Werten. Mehrfache Verletzungen einer Person sind möglich

*C2* Alkohol

Die häufigsten Verletzungsmuster in Kohorte 2 waren Hämatome der oberen (28,6 %, *n* = 10) und unteren (14,3 %, *n* = 5) Extremitäten der Untersuchten, gefolgt von Quetsch-Riss-Wunden und Kontusionen des Gesichts bei je 14,3 % (*n* = 5). Es wurden in 5,7 % (*n* = 2) Zahnverletzungen und in 2,9 % (*n* = 1) Frakturen des Gesichts festgestellt. Schädel-Hirn-Traumata ersten Grades traten bei 2,9 % (*n* = 1) der Patienten auf; intrakranielle Blutungen bei keinem der Untersuchten. Die häufigsten Frakturen befanden sich am Radiuskopf (14,3 %, *n* = 5), am Humerus und im Bereich des oberen Sprunggelenks (je 8,6 %, *n* = 3), zudem wurden bei je 5,7 % (*n* = 2) Frakturen der Hand und der Orbita diagnostiziert. Distorsionen des oberen Sprunggelenks (11,4 %, *n* = 4) und der Halswirbelsäule (5,7 %, *n* = 2) wurden ebenfalls beobachtet. Je ein Patient hatte eine traumatische Leberlazeration oder eine Milzlazeration, und 5,7 % (*n* = 2) wiesen sonographisch eine Nierenkontusion auf (Tab. 2).

Im Vergleich zwischen K1 und K2 fanden sich deutlich mehr kraniofaziale Verletzungen, inkl. Zahnschäden, bei K1 (*p* = 0,0402).

### In den Sommermonaten geschehen generell mehr Unfälle, bei alkoholisierten Fahrern geschehen diese gehäuft nachts

72,3 % (*n* = 60) der Unfälle in K1 ereigneten sich zwischen 23.00 und 7.00 Uhr und 15,7 % (*n* = 13) zwischen 15.00 und 23.00 Uhr, in 4,8 % (*n* = 4) gab es zum Unfallzeitpunkt keine Auskunft. Bei der Anzahl der Unfälle dominieren die Monate Juli bis Oktober mit 59,0 % (*n* = 49) der Unfälle, die meisten Unfälle innerhalb eines Monats (9,6 % (*n* = 8)) ereigneten sich im Oktober 2021.

Unfälle in K2 geschahen in 14,3 % (*n* = 5) zwischen 7 und 15 Uhr, in 57,1 % (*n* = 20) zwischen 15 und 23 Uhr und in 20,0 % (*n* = 7) zwischen 23 und 7 Uhr, wobei 8,6 % (*n* = 3) der Patienten hierzu keine Angaben machten. Die höchste Anzahl an Unfällen ereignete sich im August 2021 mit 14,3 % (*n* = 5). Generell wird eine höhere Unfallrate in den Monaten Juli bis Oktober mit 54,3 % (*n* = 19) der Vorfälle beobachtet.

## Diskussion

Seit der Einführung der Sharing-Dienste für E‑Scooter in Hamburg im Juni 2019 wurden auch mögliche Unfallmechanismen und Verletzungsmuster von verunfallten E‑Scooter-Fahrern bedeutsamer.

### Unfälle mit alkoholisierten Fahrern stellen ein generelles Problem dar

In dieser Studie lag der Anteil der alkoholisierten Fahrer bei mind. 32 % und liegt damit im Vergleich zu anderen Studien aus Europa eher im unteren Bereich. Interessanterweise variieren die Angaben zu diesem Kriterium in der Literatur stark. Es finden sich Angaben zwischen 5,2 und 53 % der Patienten unter Alkoholeinfluss [6, 13, 15, 19]. Einschränkend muss erläutert werden, dass sich die Zuteilung in der vorliegenden Studie in den meisten Fällen nur an anamnestischen Angaben orientieren konnte und keine objektivierbaren Messwerte wie die Blutalkoholkonzentration erhoben worden sind. Hierdurch kann eine höhere Dunkelziffer vermutet werden. Leider ließ das retrospektive Studiendesign eine nachträgliche analytische Blutalkoholbestimmung nicht mehr zu.

Die alkoholisierten Fahrer (K1) waren in dieser Studie überwiegend männlich mit knapp 70 %, während in K2 das weibliche Geschlecht dominierte. Die Gesamtkohorte (K1 + K2) entsprach dementsprechend mit 57,0 % dem bisher auch in der Literatur gezeichneten Trend, dass Unfälle von männlichen Fahrern etwas häufiger sind [4, 15, 24]. Eine Korrelation zwischen erhöhtem Risikoverhalten und dem männlichen Geschlecht, insbesondere unter Alkoholeinfluss, lässt sich vermuten und ist in anderem Zusammenhang bereits wiederholt diskutiert worden [7, 14].

Das durchschnittliche Alter von 31 Jahren in K1 und 32 Jahren in K2 unterschied sich in den in dieser Studie betrachteten Kohorten mit und ohne angegebenen Alkoholkonsum nur unwesentlich und stellt sich auch in der Literatur mit einem Durchschnittsalter zwischen 33,6 bis 37,1 Jahren ähnlich dar [6, 15]. Das niedrige Alter mag darin begründet liegen, dass ältere Personen den neuen Fortbewegungsmitteln gegenüber schon aus technischen Gründen distanziert bleiben (z. B. Notwendigkeit einer App-Installation auf einem Mobiltelefon, Verbindung zum Online-Bezahlservice).

Die Anhäufung der Unfälle in den Sommermonaten wurde auch in anderen Studien beobachtet und ist durch das bessere Wetter, das zum Fahren mit E‑Scootern einlädt, begründet [19, 23].

Die niedrigen Fallzahlen im März und April 2020 sowie ab November 2020 stimmen überein, mit den Phasen hoher Inzidenzen der SARS-CoV-2(Schweres-akutes-Atemwegssyndrom-Coronavirus Typ 2)-Pandemie, welche als Ursache für den geringeren Bedarf an E‑Scootern als Transportmittel gesehen werden kann [15, 23]. Zudem reduzierten die Sharing-Dienste die E‑Scooter in den Wintermonaten generell, woraus eine geringere allgemeine Verfügbarkeit resultierte [23].

### Nächtliche Fahrverbote?

Die Unfälle unter Alkoholeinfluss fanden alle zwischen 15 und 7 Uhr, zu 72,3 % zwischen 23 und 7 Uhr statt, während diejenigen ohne Alkoholeinfluss homogener über den Tag verteilt waren und ihren Schwerpunkt zwischen 15 und 23 Uhr zeigten. Zu ähnlichen Ergebnissen kommen auch Trivedi et al. In deren Studie traten die Auffälligkeiten v. a. zwischen 15 und 23 Uhr auf [24]. 78,5 % der Unfälle unter Alkoholeinfluss traten bei Mair et al. zwischen 22 und 6 Uhr auf [15], vergleichbar zu den hier präsentierten Ergebnissen. Auch Moftakhar et al. stellten fest, dass die meisten Unfälle und Auffälligkeiten zwischen 20 und 8 Uhr stattfanden und schlugen ein nächtliches Fahrverbot für E‑Scooter vor, welches zur Verringerung der Unfälle mit Beteiligung von E‑Scooter-Fahrern führen könnte [19]. Das primäre Ziel der flächendeckenden Versorgung mit diesen Fortbewegungsmitteln benötigt hingegen auch in den Abend- und Nachtstunden eine Verfügbarkeit, um dem Anspruch der „letzten Meile“ gerecht zu werden.

### Verletzungsmuster

Die Verletzungsmuster bei Alkoholkonsum mit einem höheren Anteil an Verletzungen des Gesichts sowie teilweise schweren Kopfverletzungen bei niedrigem Anteil an Extremitätenverletzungen zeigen reduzierte adäquate Sturzkompetenzen und Abfangreflexe an. Shiffler et al. beobachteten ebenfalls, dass bei kraniomaxillofazialen Verletzungen weniger Beteiligungen der Extremitäten zu verzeichnen waren [22]. Durch verlangsamte Reaktionszeiten bleibt die Abwehrhaltung durch Ausstrecken der Arme aus, welche normalerweise als ein natürlicher Schutzmechanismus vor Verletzungen des Abdomens, des Thorax und v. a. des Kopfes und Gesichts dient [22]. Die Schutzreflexe und adäquater Reaktionssinn wären durch Abstützen oder Schützen des Kopfes durch die oberen Extremitäten gekennzeichnet und würden somit den Kopf vor Verletzungen schützen, dabei jedoch v. a. die obere Extremität anfälliger für Verletzungen machen. Alkoholkonsum ist daher ein relevanter Risikofaktor für Gesichts- und Kopfverletzungen, da 52,6 % der alkoholisierten verunfallten E‑Scooter-Fahrer, aber nur 4,7 % der nichtalkoholisierten Fahrer diese Verletzungen aufwiesen [22]. Dies spiegelt sich ähnlich eindeutig auch in den in der vorliegenden Studie präsentierten Daten wider, in der 65,1 % (*n* = 54) der durch Alkohol beeinflussten Verunfallten und 33,3 % (*n* = 12) der zweiten Kohorte Verletzungen im Gesichts- und Kopfbereich aufwiesen. Zudem ist durch eine größere Risikobereitschaft und schlechtere Einschätzung der eigenen Fähigkeiten die Gefahr für einen Unfall unter Alkoholeinfluss prinzipiell erhöht. Schwerwiegendere Verletzungen bei Unfällen unter Alkoholeinfluss konnten auch bei anderen Verkehrsteilnehmern wie Motorradfahrern beobachtet werden [12]. Auch in Wien wurden bei Unfällen unter dem Einfluss von Alkohol vermehrt Kopfverletzungen und schwerere Verletzungen verzeichnet, diese fanden ebenfalls v. a. nachts statt. Eine nächtliche Einschränkung der Nutzung von E‑Scootern könnte das Risiko relevanter Kopfverletzungen reduzieren [19].

### Helmpflicht?

In dem in dieser Studie betrachteten Patientenkollektiv trug nach eigenen Angaben nur einer der Verunfallten einen Helm. Ähnlich niedrige Zahlen (1,7–5,2 %) werden anderswo beschrieben [6, 15, 23]. Mitchell et al. schlossen eine Helmpflicht für E‑Scooter-Fahrer in Brisbane in den Zeitraum ihrer Studie zu Verletzungsmustern ein und stellten fest, dass die Verwendung eines Helmes die Gefahr einer Kopfverletzung im Vergleich zum Nichtgebrauch signifikant senkte [18]. Störmann et al. bezogen sich ebenfalls darauf, dass Helme einen präventiven Effekt auf Kopfverletzungen haben, welche sowohl kosmetische Beeinträchtigungen durch faziale Frakturen und die hier häufig dokumentierten Zahnverletzungen als auch permanente Einschränkungen des Verletzten zur Folge haben können [23].

Da die Zahl der E‑Scooter-Nutzer und damit auch die Unfälle mit E‑Scootern steigen, sind Maßnahmen zu deren Prävention von großer Bedeutung [2].

## Limitationen

Das retrospektive Studiendesign schränkt die Datenerhebung mehrdimensional ein, da beispielsweise der Alkoholkonsum nicht näher oder bei einigen Patienten überhaupt nicht erfasst wurde. Blutproben für einen analytischen Nachweis stehen nicht mehr zur Verfügung. Des Weiteren wurden die Angaben in den elektronischen Patientenunterlagen übernommen, ohne diese weiter spezifizieren oder auf Plausibilität überprüfen zu können.

Zudem stellt das Universitätsklinikum Hamburg-Eppendorf nur eine von mehreren Anlaufstellen für verunfallte E‑Scooter-Fahrer in Hamburg dar, sodass davon auszugehen ist, dass die reale Anzahl derartiger Unfälle in Hamburg deutlich höher ist und je nach Lokalisation der Kliniken auch der Anteil der alkoholisierten Fahrer in Stadtteilen mit vielen Bars und Restaurants variieren könnte.

Die zeitlich auf zweieinhalb Jahre nach Einführung der E‑Scooter beschränkte Datenerfassung stellt ebenfalls ein Bias dar, da diese Fortbewegungsmittel an Popularität und Anzahl stetig zunehmen und damit die Wahrscheinlichkeit für Unfälle proportional steigen dürfte.

## Ausblick

Um Zusammenhänge zwischen Alkoholkonsum und Unfällen bei E‑Scooter-Fahrern weiter zu analysieren, wären eine erweiterte Recherche sowie eine fortlaufende, registerähnliche Dokumentation und Zusammenführung der Daten aus mehreren Notaufnahmen und fortführend Ballungsgebieten empfehlenswert.

Das voraussichtlich weiterhin hohe oder sogar steigende Angebot an E‑Scootern und die damit einhergehende zunehmende Nutzung machen eine Aufklärung der Fahrer über die Gefahr von Unfällen, insbesondere unter Alkoholeinfluss, notwendig. Eine benötigte Bestätigung in der App des Sharing-Dienstleisters, nicht unter Alkoholeinfluss zu fahren, wie sie beispielsweise schon durch den Verleihservice LIME gefordert wird, ist eine sinnvolle Maßnahme [13]. Diese Schritte sollten das Bewusstsein der Nutzer für gesundheitliche und rechtliche Folgen von alkoholisierten Fahrten fördern. Die Einführung einer Helmpflicht wäre sinnvoll, um häufig auftretende Kopf- und Gesichtsverletzungen zu vermeiden oder in ihrer Schwere zu reduzieren. Ebenso wäre ein nächtliches Fahrverbot in Stadtteilen mit vielen Bars und Restaurants eine Option, die konsequent den Anteil alkoholisierter Unfallfahrer vermindern würde, aber auch alkoholnüchternen Interessenten dann die Möglichkeit einer schnellen, mobilen und modernen Methode der elektronischen Fortbewegung nehmen würde.

## Fazit für die Praxis

- Unfälle von E‑Scooter-Fahrern ereignen sich v. a. nachmittags, abends und nachts.
- Alkoholisierte Verunfallte sind häufig Männer mit einem durchschnittlichen Alter von 31 Jahren.
- Verletzungen des Gesichts und des Kopfs sind unter Alkoholeinfluss häufiger nachzuweisen und in ihrer Intensität schwerer ausgeprägt.
